# A 2021 Update on Syphilis: Taking Stock from Pathogenesis to Vaccines

**DOI:** 10.3390/pathogens10111364

**Published:** 2021-10-21

**Authors:** Giorgio Tiecco, Melania Degli Antoni, Samuele Storti, Valentina Marchese, Emanuele Focà, Carlo Torti, Francesco Castelli, Eugenia Quiros-Roldan

**Affiliations:** 1 Unit of Infectious and Tropical Diseases, Department of Clinical and Experimental Sciences, ASST Spedali di Brescia, University of Brescia, 25123 Brescia, Italy; g.tiecco@unibs.it (G.T.); m.degliantoni@unibs.it (M.D.A.); s.storti@unibs.it (S.S.); v.marchese@unibs.it (V.M.); emanuele.foca@unibs.it (E.F.); francesco.castelli@unibs.it (F.C.); 2 Infectious and Tropical Disease Unit, Department of Medical and Surgical Sciences, “Magna Graecia” University of Catanzaro, 88100 Catanzaro, Italy; torti@unicz.it

**Keywords:** syphilis, sexually transmitted infections, HIV infection, pathogenesis, vaccine

## Abstract

In 2021 the scientific community’s efforts have been focused on solving the back-breaking challenge of the COVID-19 pandemic, but sexually transmitted infections (STI) are still one of the most common global health problems. Syphilis is a systemic disease caused by the spirochaete Treponema pallidum (TP) and is one of the oldest known diseases. Its incidence has increased in the last few years and syphilis still remains a contemporary plague that continues to afflict millions of people worldwide. Despite research improvements, syphilis pathogenesis is not completely clear; clinical presentation is very heterogeneous and the diagnosis can sometimes be difficult. Furthermore, few therapeutic options are available, and a vaccine has not been found yet. In this review, we describe the most recent evidence concerning the clinical manifestation, diagnosis, treatment and vaccine prospectives for this disease.

## 1. Introduction

In 2021 the scientific community’s efforts have been focused on solving the *back-breaking* challenge of the COVID-19 pandemic, but *sexually transmitted infections* (STI) are still one of the most common global health problems.

Syphilis is a systemic disease caused by the spirochaete *Treponema pallidum* (TP) and is one of the oldest known diseases for which curative and inexpensive treatment is available. Without animal reservoirs, theoretically, syphilis should be an eradicable disease, but to date, multiple concerted efforts to eliminate syphilis have failed, such as the *Syphilis Elimination Effort* launched by United States *Center for Disease Control and Prevention* (CDC) in 1999 [1]. 

The complex natural history of syphilis reflects the invasiveness and immune evasiveness of TP. The infection does not lead to immunity against reinfection and repeated episodes of syphilis occur, predominantly in *men who have sex with men* (MSM) with a high rate of partner change [2]. Moreover, HIV infection may modulate the clinical presentation and the clinical and serologic response to syphilis treatment [3,4,5,6]. 

In 2017, the annual rate of primary and secondary syphilis in the United States was 11% higher than in 2016: more than 60% of infected individuals were MSM, of whom 46% were *people living with HIV infection* (PLWH) [7]. The rate continued to rise consecutively through 2019 [8], and recent data estimates a global pooled syphilis prevalence among MSM of 7.5% (95% CI 7.0–8.0%) over the last 20 years [9]. Preliminary 2020 data suggest that this trend continued in 2020, despite the COVID-19 pandemic and the decrease of syphilis testing and treatment during this period [10]. 

Syphilis facilitates both HIV transmission and HIV acquisition. The impact of syphilis infection on the risk of HIV-RNA elevation and decline in CD4+ reflects the complex interplay between these two diseases [11]. 

Although syphilis is a very old disease, its pathogenesis is not fully understood, diagnosis may be difficult and, lastly, few therapeutic or prophylactic options are available at the moment. 

## 2. Etiology and Pathogenesis

TP is a member of the family *Spirochaetaceae*, and genus *Treponema*, which includes four human pathogens (*T. pallidum pallidum*, *T. pallidum endemicum*, *T. pallidum pertenue* and *T. pallidum carateum*) and at least six human nonpathogens. 

TP is characterized by a rare outer membrane and a slow replication rate (about 33 h) with a dynamic regulation of TP genes, which is important for its successful colonization, dissemination, and invasion in hosts [12,13]. *TP repeat genes* (*Tpr*) encode proteins that mediate attachment to host tissue and function as porins. *Tpr* proteins are immunogenic and one of them, *Tpr K*, has been described as being a target for opsonic antibodies. *Tpr K* differs in seven discrete variable regions and antibodies to these variable regions offer only homologous protection and not against heterologous strains. Antigenic variation through gene conversion during infection seems to be the mechanism by which TP avoids the host immune response, allowing for prolonged infection and persistence even in the case of a robust host response [14]. TP is rich in transport proteins, and this may have a central role in antibiotic penetration through the bacterial membrane [15]. 

TP is presumed to penetrate through small skin lesions, but the exact mechanisms by which TP enters cells is not known. The invasion of endothelial cell monolayers and intact membranes are the main virulence features of the bacteria [16]. 

TP is one of the few pathogens capable of crossing specialized endothelial barriers such as the retinal, placental, and blood-brain barriers. This seems to occur through the TP protein Tp0751, a host-binding vascular adhesin, also referred to as *pallilysin*, that interacts with microvascular and macrovascular endothelial cells (including cerebral endothelial cells) through an endothelial receptor named LamR [16]. 

Although widely discussed, neuroinvasive strains are hypothesized: TP 14a/a and 14d/f. Those strains have been described as possessing a greater potential of neuroinvasion, and they may be better able at evading the host immune responses in the central nervous system (CNS) when compared to other strains in both humans and rabbits [17]. 

Whether HIV infection alters the natural history of syphilis or vice-versa remains controversial. TP can induce the expression of CCR5 (the major co-receptor for HIV entry) on monocytes in syphilitic lesions, enhancing the transmission of *macrophage-tropic* HIV-1 [18]. However, the impact of syphilis on CD4+ cells or HIV viremia seems marginal [19].

## 3. Clinical Manifestation

Syphilis clinical manifestations are so wide and heterogeneous that a classification based on clinical stages, chronologically beginning with the onset of a chancre, was needed. Nevertheless, stages might overlap (especially in PLWH) and the definition of early and late syphilis is still debated:

⮚ According to the Centre for Disease Prevention and Control (CDC), early syphilis is defined as syphilis acquired <1 year previously, otherwise it is considered late syphilis.
⮚ According to the World Health Organization (WHO), early syphilis is defined as syphilis acquired <2 years previously, otherwise it is considered late syphilis.

Early syphilis includes:

⮚ Primary syphilis: A *chancre* is a solitary skin indurated, non-exudative, painless ulcer that follows the acquisition of TP. Multiple and painful chancres, deeper and slower to resolve, have been described more commonly in PLWH [20,21].
⮚ Secondary syphilis is a systemic disease due to bacteraemia and it follows primary syphilis within weeks to a few months [22]. Secondary syphilis can produce a wide variety of signs and symptoms with a broad differential diagnosis [23,24] (Table 1). In PLWH, the early stages of syphilis overlap more frequently.

▪ *Malignant lues*, a severe cutaneous *ulcero-nodular* form, has also been described more frequently in PLWH and has been related to a defective cell-mediated immune response [25].
▪ TP may infect the CNS at any stage. Although a significant proportion of patients with early syphilis and evidence of cerebrospinal fluid (CSF) abnormalities are asymptomatic (*asymptomatic neurosyphilis*), TP may disseminate to the central nervous system within hours to days after inoculation [26]. In PLWH at high risk for laboratory-defined neurosyphilis, cognitive complaints are not a good indicator of cognitive impairment [27]. Early neurosyphilis may also include symptomatic forms as *meningeal/meningovascular* forms and *ocular/otic* syphilis [6,28]:
  ▪ *Ocular syphilis* may occur without other CNS manifestations and has been documented to affect almost every structure of the eye, resulting in blindness, especially in PLWH with a CD4+ count < 200 cells/microL [29].
  ▪ *Otosyphilis* can manifest with a variety of audiovestibular symptoms (hearing loss is the main one). TP may also affect the eighth cranial nerve, the cochleovestibular apparatus, or the temporal bone [30].

⮚ Early latent syphilis refers to the asymptomatic period between primary and secondary or late syphilis. Up to 24% of untreated patients suffer from secondary lesion relapses (more frequently among PLWH) during the first year of infection [28]. For this reason, a 1-year cut-off period is frequently used to classify early and late syphilis [28].

Repeated episodes of syphilis in PLWH are mostly asymptomatic [4]. Patients with > 3 episodes of syphilis were more likely to have early latent syphilis than primary or secondary syphilis at the index visit compared to those with < 2 episodes [31]. 

Late syphilis occurs in 25% to 40% of patients with untreated syphilis. Clinical events may appear at any time, 1 to 30 years after the primary infection [6].

⮚ Tertiary syphilis includes patients with late syphilis that show symptomatic manifestations. PLWH may progress to tertiary syphilis more rapidly than HIV-uninfected patients [32]. The more frequent forms are:
▪ *Cardiovascular syphilis* classically involves the ascending thoracic aorta, resulting in a dilated aorta and aortic valve regurgitation. It is thought to be a consequence of vasculitis in the vasa vasorum. Many diagnoses of syphilitic aortitis are invariably obtained or suspected on histopathological examination [33]. Syphilis may also involve coronary arteries, resulting in coronary artery narrowing and thrombosis [34].
▪ Gummatous syphilis presents as ulcers or heaped up granulomatous lesions (*gummas*). Several cases of *gummas* involving internal organs (*visceral gummas*), including the CNS, have been reported in PLWH [35].
▪ *Meningovascular neurosyphilis* results from inflammation of large- to medium-sized arteries of the brain or spinal cord [36]. Neurosyphilis and HIV should be investigated in young patients with cerebral infarction.
▪ *Parenchymatous neurosyphilis* is characterized by the destruction of cortical CNS parenchyma, clinically mimicking a mental disorder or dementia. When the infection involves the posterior column and nerve roots, it is known as *Tabes dorsalis*. It is estimated that 1.5–9% of cases of syphilis are complicated by *Tabes dorsalis* and is more commonly observed in MSM and Black people [37].
▪ *Late Neurosyphilis*, includes a paucisymptomatic form (with alterations in cerebrospinal fluid) that is present in about 15% of patients originally diagnosed as having latent syphilis and in 12% of those with cardiovascular syphilis. In one study of 117 PLWH diagnosed with neurosyphilis, approximately 33% were asymptomatic [38]. Without treatment, it evolves into symptomatic neurosyphilis in 5% of cases [28].

Latent late syphilis refers to the period when a patient infected for >1 year with TP (as demonstrated by serologic testing) has no symptoms.

## 4. Diagnosis

Diagnostic testing for syphilis should be performed on both symptomatic and asymptomatic patients at high risk of acquiring the disease or transmitting it to others. CDC suggests annual screening for sexually active MSM, although testing as often as every three months may increase the early detection of syphilis [6,39]. 

In PLWH, guideline panels recommend screening at the initial visit, and then annually for those who are sexually active [40]. More frequent screening (every three to six months) is recommended for those with multiple sex partners and those who engage in unprotected intercourse or have sex in conjunction with illicit drug use [36].

### 4.1. Indirect Methods

Serologic testing is the primary tool for diagnosing syphilis [40]. Two types of serologic tests are required for diagnosis. 

▪ Nontreponemal tests (NTT) (also known as *reagin tests*) are based upon the reactivity of serum from infected patients to a *cardiolipin-cholesterol-lecithin antigen*. Traditionally, they have been used for initial syphilis screening (*traditional algorithm*) (Table 2) and include RPR (*Rapid Plasma Reagin*), VDRL (*Venereal Disease Research Laboratory*) and TRUST (*Toluidine Red Unheated Serum Test*). The results are quantitative and reported as a titer of antibodies. According to the traditional approach, a negative result excludes the diagnosis of active syphilis and no further testing is needed. These tests show a 62–78% sensitivity for primary syphilis, 97–100% for secondary and 80–100% for early latent syphilis [38]. The sensitivity of NNT is lower for late syphilis whereas, in early syphilis, a false negative might present in 30% of cases as a consequence of testing before the development of antibodies [41].

Biological false positive, although uncommon, must be considered during pregnancy or autoimmune illnesses as well as other infectious diseases. Currently, NTT are manually performed and several attempts to automate these tests have been described [42].

Monitoring of nontreponemal test titer should be done using the same test and it is indicated to:

- Evaluate treatment response (Table 3)
  Titer tends to wane over time even without treatment, but successful therapy accelerates the pace of antibody decline.
  *A serological cure* is defined as a *seroconversion* (from positive to negative) or as a *4-fold* (or two dilutions) decline in NNT antibody titer 6 to 12 months after therapy for early syphilis and 12 to 24 months for late syphilis [44]. A *4-fold* or greater titer decline is generally associated with younger age, higher baseline nontreponemal titers, and earlier syphilis stage. *Treatment failure* is defined as a ≥4-fold rise in nontreponemal titers after treatment in the absence of reinfection [44]. Patients correctly treated, with a ≤4-fold titer decrease and unlikely to have a new infection, are known as *serological non-responders*. *Serofast* status concerns patients with a persistently reactive NTT despite adequate treatment without seroconversion after an initial ≥4-fold decline [44,45].
- Evaluate reinfection
  An individual with a previous serological cure might be considered as “re-infected” if a new *seroconversion* (from negative to positive) or a *4-fold* or *greater increase* in antibody titer occurs (Table 3 and Table 4).

▪ Treponemal tests (TT) are based on the detection of antibodies directed against specific treponemal antigens and they have traditionally been used as confirmatory tests for syphilis when NTT are reactive [6]. Usually, their results are reported qualitatively. They include FTA-ABS (*fluorescent treponemal antibody absorbed*) and TP-PA (*TP particle agglutination*) which detect both IgG and IgM, or EIA (*enzyme immunoassay*) and immunoblot able to detect IgG or IgM. These tests are increasingly used as an initial screening test for syphilis rather than as confirmatory tests (*reverse algorithm*; Table 2). Once a patient has a positive TT, this test usually remains positive for life. In other words, quantification of TT titer is not useful in diagnosing reinfections or monitoring disease after treatment. TT false positives may be encountered on other occasions, including different spirochaetal infections [43].
  The choice of screening test depends on resource availability, either economical or human, since some TT are automatized, such as ELISA/EIA/CLIA. Moreover, disease prevalence should also be considered, as in low prevalence settings, TT may result in very low positive predictive value [40].

In PLWH, serologic testing for syphilis can be interpreted in the same manner regardless of HIV status. However, clinicians should be aware of the unusual serologic responses in PLWH who have syphilis [46]. In primary and, less commonly, in secondary syphilis, a delayed appearance of *sero-reactivity* is common and false-negative tests might be associated both with the *Prozone Effect* as well as with the advanced immunodeficiency of the patient (it is thought to reflect B-cell failure during late-stage HIV infection) [46,47]. 

### 4.2. Direct Methods 

Since TP has always been taught to be non-culturable, currently the organism must be identified through direct visualization or detection in clinical specimens. *Darkfield microscopy* and *direct fluorescent antibody* testing can be used to detect the organism; however, neither of these tests are routinely available in clinical settings [40]. A recent study achieved a reproducible multiplication of TP *subsp. pallidum* using a long-term in vitro culture. Although the potential diagnostic implications are currently limited, this may lead to a better understanding of TP physiology, structure, gene expression, regulatory pathways, pathogenesis, immunologic properties, and antimicrobial susceptibility [48].

Some laboratories have developed polymerase chain reaction (PCR) tests to detect TP DNA from clinical specimens (*tpp47* and *polA* are the more frequently amplified targets) with variable sensitivity and specificity depending on specimens (lower in blood and cerebrospinal fluid) [49]. Currently, they are not approved by the *Food and Drug Administration* (FDA), but PCR is becoming widely used in some jurisdictions thanks to the encouraging results in detecting early primary syphilis lesions before the development of any serological marker [50]. Moreover, a recent study states that DNA tests with oral and anal swabs, also in the absence of oral or anal lesions, may reduce the time to diagnosis, preventing progression to the secondary stage and improving syphilis control [51].

Point-of-care (POC) tests, which can be performed at clinical visits, have been hypothesized to have a positive effect on shortening the duration of the infection, because patients can be treated before they leave the clinical encounter. Those tests should be highly sensitive and specific and inexpensive. Most of the available syphilis POC tests detect antibodies specific to TP, and they perform reasonably well in either whole blood or serum specimens, with sensitivities ranging from 74% to 99% and specificities ranging from 94% to 99%, but the main challenge is their inability to differentiate prior treated infections from current infections.

### 4.3. Neurosyphilis Diagnosis 

There is no consensus for neurosyphilis diagnosis but if the diagnosis of neurosyphilis is being considered, additional testing on cerebrospinal fluid (CSF) should be performed [52,53]:

▪ *CSF Analysis*: Elevated white blood cells and proteins in CSF are often seen in neurosyphilis but are usually considered nonspecific findings. A CSF cell count >5 cells/microL is suggestive of neurosyphilis. The threshold is higher in PLWH since those may have a CSF pleocytosis due to HIV itself. For this reason, a CSF cell count >20 cells/microL is suggestive of neurosyphilis in PLWH [54].
▪ *Non-Treponemal Test*: RPR and VDRL in CSF are 100% specific for diagnosis of neurosyphilis but their sensitivity is poor (CSF VDRL is 49–87% sensitive and RPR 51–82%) [41].
▪ *Treponemal Test*: CSF FTA-ABS test has been suggested to have a strong negative predictive value and neurosyphilis is highly unlikely if negative [55].

The definition of asymptomatic neurosyphilis is quite contentious. Whether or not lumbar puncture should be carried out in patients with no neurological involvement remains controversial. CSF examination might be warranted in *serological non responders* and *serofast* patients [40]. Many experts believe that most patients with HIV and syphilis deserve CSF examination regardless of symptoms, especially those with a CD4+ cell count < 350 cells/microL and an RPR > 1:32 [54,56]. 

### 4.4. Syphilis Reinfections Diagnosis 

It is difficult to distinguish syphilis reinfection from disease relapse since the diagnosis depends on clinical findings of syphilis and a *4-fold* increase in NTT titre (Table 4). 

Syphilis infection leads only to partial immunity to reinfection. Thus, subsequent episodes of syphilis may not present in the same way as the initial episodes [57]. Moreover, in settings where syphilis is regularly screened for high-risk persons, syphilis is more likely to be diagnosed before the development of clinical symptoms, simply because of changes in titres of non-treponemal tests [58]. 

Data regarding serological response in patients with repeated episodes are discordant. Patients with syphilis reinfection had higher VDRL/RPR and TPPA serum titres but lower IgM serum titres than patients with one syphilis episode [59]. Additionally, each additional episode of syphilis results in a slightly attenuated immune response [3]. As a result, the diagnosis of repeat syphilis may be both under- and over-diagnosed. In the very near future, the routine use of PCR tests for TP DNA might be the solution for this issue.

## 5. Treatment

No controlled clinical trials are available to optimize the treatment of syphilis. Recommendations are mainly based on laboratory results, expert opinions, clinical cases and experience. Penicillin G benzathine (PGB) is the treatment of choice for syphilis and is preferred to short-acting penicillin. However, the dosage, formulation, and duration of treatment depend upon the stage of disease and whether the infection involves “*protected sites*” that “hide” *T. pallidum* (ocular structures or CNS). Scarce data are available for alternative treatments (Table 5). 

A nontreponemal serologic test should be obtained just before initiating therapy (ideally, on the first day of treatment) to establish the pre-treatment titre. This test is critical to establish the adequacy of the post-treatment serologic response [60].

▪ Treatment of Early Syphilis (primary, secondary, and early latent syphilis) [6,28,40]
  The standard therapy based on a single intramuscular (IM) dose of PGB (2.4 million UI) maintains this serum concentration up to 21 days. Increasing the dose does not clear treponemes more quickly.
  Alternative regimens are typically administered to patients who are unable to take penicillin or when it is unavailable. *Doxycycline* or *Tetracycline* are both employed, with a serologic response in 82.9% of patients [61].
  *Ceftriaxone* is a promising alternative to PGB with a good CNS penetration, long half-life that enables once-daily dosing and efficacy similar to PGB, but there are limited clinical data and the optimal dose and duration of treatment have not been defined yet [62,63]. *Azithromycin* is an alternative, although generally not recommended due to the rapid emergence of macrolide resistance in TP.
  The molecular basis for macrolide resistance is mediated by point mutations in the TP *23S* ribosomal RNA gene at nucleotide positions 2058 and 2059 in both copies of the 23S rRNA genes. A2058G mutations conferring macrolide resistance are more common than A2059G [64,65]. To date, there have been no reports of strains possessing both mutations. Macrolide-resistant TP with the A2058G mutation is now present in several areas of the USA, Canada, Europe, and China, with a wide range of prevalence (from 16% in Canada, 85% in France and >90% in China) [66,67,68].
  PLWH with syphilis should be treated as HIV-uninfected patients, although they may be at increased risk for treatment failure and may be more likely to progress to neurosyphilis [69,70]. Several studies have evaluated enhanced therapy with additional doses of PGB and found no additional benefit [71,72]. There are limited data on the efficacy of alternative regimens in PLWH; therefore, close monitoring of these patients must be carried out [73].
▪ Treatment of Late syphilis (late latent syphilis, neurosyphilis) [6,28,40]
  Extended treatment is needed as the duration of the infection increases (more relapses have been seen in later stages after short courses of treatment). During late syphilis, a cerebrospinal fluid examination before initiation of therapy is necessary to investigate neurosyphilis.
  If neurosyphilis is excluded, the “*treponemicidal”* level of PGB must be maintained for 21 days and 2.4 million UI IM once weekly for 3 weeks is the standard therapy. If a patient misses a dose or more than 14 days have elapsed since the prior dose, the course should be reinitiated. For those with cardiovascular disease, antibiotic therapy does not reverse the clinical manifestations of syphilis, but it may halt the progression of the disease.
  Patients with neurosyphilis should generally be treated with intravenous (IV) therapy, because the dose of IM PGB that is administered for other stages of syphilis does not produce measurable CSF levels of the drug. IV therapy should be administered also to patients strongly suspected of having CNS syphilis, even if they have a nonreactive CSF-VDRL. Alternatives to these therapeutic regimens are poorly studied in this setting. Treatment based upon CSF results, in the absence of neurological involvement, has not been associated with improved clinical outcomes, but may mitigate subsequent cognitive decline [27].
  There is no consensus on the management of *serofast* or *serological non-responders*; a recent study among HIV-uninfected patients with a poor serological response after an appropriate treatment shows no additional benefit in retreatment [74].

*Jarisch Herxheimer reaction* (JHR) is a transient clinical phenomenon that occurs in patients infected by spirochetes, including TP, within 24 h of antibiotic therapy. It usually manifests as fever, rigors, nausea and vomiting, headache, tachycardia, hypotension, and exacerbation of skin lesions and it is important to distinguish it from antibiotic allergic reactions. Antibiotics mostly associated with the development of JHR are penicillins, tetracyclines and erythromycin, and rarely with cephalosporins and fluoroquinolones. This reaction can be expected in 55% to 95% of primary or secondary syphilis [75]. It is, however, very rare in late syphilis, but often unrecognized and underreported [75]. In PLWH, JHR occurs in 35–56% of treated patients, and for each *2-fold* increase in RPR titer, the risk of JHR grows by 19%, suggesting that a higher load of spirochetes in the early stages of syphilis increases the risk of JHR [76]. In patients with neurosyphilis and HIV encephalitis, JHR has been described to be more severe and prophylaxis with anti-inflammatory agents could be employed [76,77]. 

### Follow Up after Treatment 

Patients should be monitored both clinically and with laboratory testing to ensure an appropriate response to therapy. 

Clinical follow-up post treatment is recommended in patients with early syphilis to assess the resolution of symptoms. However, for patients with late-stage *cardiovascular* or *noncutaneous gummatous* disease, a significant change in symptoms is unlikely. For individuals with symptomatic neurosyphilis, serial neurological examinations should be performed every six months after treatment [54]. 

Serological follow-up with *Nontreponemal titers* every six months (every three months in PLWH) after treatment is recommended until *serological cure*. PLWH have a slower decline of NTT and seroreversion is observed in about half of treated patients, although it might take years to occur [46]. 

Additional treatments do not provide short-term serological benefits beyond watchful waiting. In cases of neurosyphilis with the presence of CSF pleocytosis or a positive CSF serologic test before treatment, CSF examinations should be repeated every six months until the cell count becomes normal. Resolution of the CSF pleocytosis is the most sensitive indicator of effective treatment in neurosyphilis. 

Many studies have evaluated the association between HIV infection and poor serologic response following syphilis treatment: this seems to be less frequent in patients with high CD4+ cell counts, confirming the importance of those cells for an appropriate serological response [46]. Both clinical and serological follow up in reinfections are more difficult because multiple episodes of syphilis seem to attenuate clinical and laboratory manifestations of *TP* infection. Furthermore, the detection of TP DNA in blood samples was significantly lower in those with previous syphilis [31]. However, further studies are needed. 

## 6. Syphilis Prevention and Vaccines 

The high incidence of syphilis worldwide, despite available and inexpensive treatment, emphasizes the need to seek an alternative approach for syphilis control. Combined strategies of public health (i.e., implementation of screening and treatment campaigns) and vaccine development should be encouraged. 

There are few studies regarding the use of chemoprophylaxis for syphilis. A randomized pilot study showed the effectiveness of a potential post-exposure prophylaxis (PEP) with daily doxycycline for syphilis in MSM [78], revealing a potential benefit for this population. More recently, the ANRS IPERGAY trial confirmed the efficacy and safety of PEP with a single 200 mg dose of doxycycline (taken within 24 h after sex) in MSM taking pre-exposure prophylaxis (PrEP) for HIV prevention. The occurrence of a first episode of syphilis in participants taking PEP was lower than in those not taking PEP (HR 0.27; 95% CI 0.07–0.98; *p* = 0.047) [79]. Although the undoubted benefits of this practice, it is reasonable to be aware of the risks for the onset of antimicrobial-resistant strains. 

Notwithstanding this, vaccines might be the ideal tool of prevention because of their long-lasting protection, but syphilis vaccine development presents several challenges. The limited availability of long-term culture systems [48] and the TP surface-exposed antigen shortage are the main reasons why a viable candidate is still unavailable and probably years away from discovery. 

Several syphilis candidates have been investigated over the past few years, including whole-cell inactivated, live attenuated or genetically engineered vaccines [80]. 

Among them, members of the TP repeat (*TPr*) family of paralogs, such as *TprK* (TP0897), have emerged as promising candidates in vaccine development studies. Although *TprK* is antigenically unreliable and known to play a role in immune evasion, the structural model of the protein predicts three conserved surface-exposed loops in the *TprK* NH2-terminus. Immunization of rabbits with a recombinant NH2-terminal fragment of *TprK* purified under denaturing conditions attenuated cutaneous lesion development and progression to ulceration. Additionally, the treponemal burden challenge sites were significantly reduced compared to controls [81,82].

A whole-cell bacterial vaccine might be more effective than a recombinant antigen-based vaccine and, accordingly, a group of scientists decided to employ this approach to present TP antigens in their native conformation within a spirochete membrane compartment similar to that of the syphilis pathogen. This heterologous antigen presentation system used *Borrelia burgdorferi* engineered to express TP genes. A limited amount of the TP Tp0435 lipoprotein, previously believed to be exclusively periplasmic, was considered to also possibly gain surface exposure in both *B. burgdorferi* strains—this was then confirmed in TP. Partial protection was observed in rabbits immunized with *B. burgdorferi* expressing Tp0435 and immunity was not protective [83].

Lastly, a plasmid DNA encoding TP flagellin *FlaB3* emerged as a candidate for vaccine development, evaluation of immunogenicity and protection against dissemination, showing a significant reduction in the bacterial burden in the blood, liver, spleen and testicles of immunized rabbits [84]. 

Further studies are needed to assess the proper means of immunization, the type of immune response elicited, and the duration of immunity [85].

## 7. Conclusions

The resurgence of syphilis has revived interest both in developing and developed countries—especially in PLWH. Syphilis still presents several challenges: identifying reinfections, monitoring therapy appropriately with serological tests and developing a correct approach to asymptomatic neurosyphilis. Biomedical interventions such as PrEP, PEP and vaccines for syphilis are not available and condom use remains the cornerstone of prevention for syphilis. 

Future priorities include validating rapid and direct detection methods for syphilis (*point of care test*), finding alternative treatment regimens, and developing more effective means of prevention.

## 8. Research Strategy and Selection Criteria

References for this review were identified from PubMed, Embase and Cochrane with the research terms: “*syphilis*”, “Treponema”, “*HIV*”, “*treatment*”, “*diagnosis*” and “*vaccine*” with several combinations. Only papers in English were included. The final reference list was generated based on timeline, originality and relevance to the scope of this Review.

## Figures and Tables

**Table 1:** Signs and symptoms of secondary syphilis.

**Constitutional Symptoms**	Diffuse symmetric macular or papular rash, condyloma lata, alopecia, fever, headache, myalgias, weight loss and adenopathy
**Musculoskeletal**	Synovitis, osteitis and periostitis
**Hepatic**	High serum alkaline phosphatase level
**Renal**	Albuminuria, nephrotic syndrome, glomerulonephritis, nephritis membranous glomerulonephritis and diffuse endocapillary glomerulonephritis
**Neurological**	Meningitis, cranial nerve deficits or stroke and myelitis
**Otic**	Hearing loss, tinnitus, vertigo
**Ophthalmic**	Uveitis, retinal necrosis and optic neuritis

**Table 2:** Differences between the traditional approach and reverse approach for syphilis diagnosis.

	Traditional Approach	Reverse Approach
Definition	*Non Treponemal Test* first	*Treponemal test* first
Pros	Easy to perform Ability to be quantified	High sensitivity in early stages
Cons	High False Negative rate especially in early stage of disease High risk of biological False Positive	High False Positive rate in area with endemic, non-venereal treponematoses [43]
When to use	Low prevalence areas of syphilis	High prevalence areas of syphilis

**Table 3:** Syphilis: serological definitions after treatment.

**Serological cure**	Seroconversion (from positive to negative) or a ≥*4-fold* decline in nontreponemal antibody titers
**Treatment Failure**	A ≥*4-fold* rise in nontreponemal titers after treatment in the absence of reinfection
**Serologic-nonresponse**	A *≤4-fold* decrease in nontreponemal titers after an appropriate treatment in the absence of reinfection
**Serofast status**	A reactive nontreponemal test despite adequate treatment without seroconversion after an initial ≥*4-fold* decline in nontreponemal titers
**Reinfection**	A new seroconversion (from negative to positive) or a ≥*4-fold* rise in nontreponemal titers although a previous serological cure

**Table 4:** Serological tools for syphilis diagnosis.

	Treponemal Test +	Treponemal Test −
Non Treponemal test +	New Infection. If a previous history of syphilis is present, consider non treponemal test titer to evaluate *reinfection*, *treatment failure* or *serofast status.* If no symptoms are present, consider Latent Syphilis.	Non treponemal test False Positive
Non Treponemal test -	Serological responders. If no syphilis has been treated before and no signs or symptoms are detectable, consider repeating treponemal test (*Late Latent Syphilis*) If no syphilis has been treated before, but clinical findings are suitable with syphilis diagnosis, consider repeating non treponemal test and, eventually, performing a cerebrospinal fluid analysis (*Early latent syphilis*).	Consider other diagnosis

**Table 5:** Recommended regimen for syphilis treatment [6,28,40].

	Early Syphilis	Latent Syphilis
First Line	- Benzathine Penicillin G 2.4 MIU IM single shot	If neurosyphilis is excluded: - Benzathine Penicillin G 2.4 MIU IM once/week for 3 weeks If neurosyphilis is not excluded: - Benzathine Penicillin G 18–24 MIU IV qd (as 3–4 MIU every 4 h) for 10–14 days
Alternative	- Procaine Penicillin 0.6 MIU IM qd for 10–14 days - Doxycycline 200 mg po qd for 10–14 days - Ceftriaxone 1 g IV qd for 10–14 days	If neurosyphilis is excluded: - Procaine Penicillin 0.6 MIU IM qd for 17–21 days - Doxycycline 200 mg po qd for 21–28 days If neurosyphilis is not excluded: - Procaine Penicillin 1.2–2.4 MIU IM qd + Probenecid 500 mg qid for 10–14 days - Ceftriaxone 1–2 g IV qd for 10–14 days

Acronyms: MIU (Million International Units); IM (intramuscular injection); IV (intravenous injection); po (per os); qd (quaque die).

## Data Availability

Not applicable.
